# Cutaneous metastasis from cervical cancer to the scalp and trunk: a case report and review of the literature

**DOI:** 10.1186/s13256-023-04171-x

**Published:** 2023-10-19

**Authors:** Ying Dai, Yufei Zhang, Xue Ke, Yunqin Liu, Chunbao Zang

**Affiliations:** Department of Radiation Oncology, The First Affiliated Hospital of USTC, Division of Life Sciences and Medicine, University of Science and Technology of China, Anhui Provincial Cancer Hospital, Hefei, 230031 Anhui China

**Keywords:** Cervical cancer, Cervical cancer metastasis, Cutaneous metastasis, Case report

## Abstract

**Background:**

An estimated 119,300 new cases of cervical cancer occur annually in China, accounting for 372,00 deaths. Cutaneous metastasis from cervical cancer is a rare event, with an incidence of 0.1–1.3% and typically a preterminal occurrence. Scalp metastasis from cervical cancer is exceptionally anecdotal, with only a dozen examples well documented.

**Case presentation:**

The patient is a 33-year-old Chinese woman who was diagnosed with International Federation of Gynecology and Obstetrics stage IVB cervical cancer in November 2021. From December 2021 to April 2022, the patient was enrolled in the clinical trial of sintilimab combined with chemotherapy and radiotherapy for treatment of stage IV cervical cancer and underwent six cycles of immunotherapy and chemotherapy (sintilimab plus paclitaxel liposome and cisplatin). Treatment was well tolerated and led to a partial response. The masses adjacent to the spine and iliac bone was largely reduced. Thus, radiotherapy of the metastatic residues was carried out and followed by radiotherapy to the primary tumor at the cervix uteri. However, by the time of the radiotherapy completion in October 2022, the patient noticed painless nodules at the left scapular region and the right hypochondrium. The following month, more nodules occurred on the scalp and trunk, including the left axilla, anterior abdomen, and left back, along with a lesion invading the sternum that caused acute bone pain. The cutaneous masses were white, discrete with a rubbery consistency, and fixed to the skin. Several nodules increased in size and eventually ulcerated. Fine‑needle aspiration cytology of the left back swellings revealed metastatic squamous cell carcinoma, P16 positive. No visceral or brain metastasis was observed at this point.

**Conclusions:**

Cervical cancer metastases to the scalp are extremely uncommon. When a scalp metastasis is present, it might be the only symptomatic sign of disease progression or widespread metastatic lesions. So far, there is no clear guideline regarding skin metastases treatment. Such skin lesions warrant a thorough radiologic and pathologic workup to form a comprehensive management plan.

## Introduction

Despite advances in prevention, screening, diagnosis, and treatment over the past decades, cervical cancer (CC) still ranks as the fourth most diagnosed cancer in women [1, 2]. An estimated 604,127 new cases of cervical cancer occurred worldwide annually, and 119,300 in China [3]. Cervical cancer accounted for 341,831 cancer deaths globally and 37,200 deaths in China and continues to be a major problem of public health [2, 3].

The most common sites of distant metastasis of cervical cancer are lungs, bone, and liver, while the less frequent locations of spreading are the bowel, adrenal gland, spleen, and brain [1]. Cutaneous metastasis from cervical cancer is a rare event with an incidence of 0.1–1.3% [4, 5]. The three major regions of skin metastasis in females were abdominal wall, vulva, and anterior chest wall [5]. Metastasis to the scalp from internal malignancy could only be seen in around 4% of cases [6]. In men, the primary tumor with scalp metastasis was often in the lung or kidney, while in women, the breast was the most frequent primary site [6]. Scalp metastasis from cervical cancer is exceptionally anecdotal, with only a dozen examples well documented in the literature [7–18]. Here, we report a case of cervical carcinoma with multiple metastases to the scalp and the trunk that, to our best knowledge, is the first report in mainland China.

## Case presentation

**Fig. 1:**
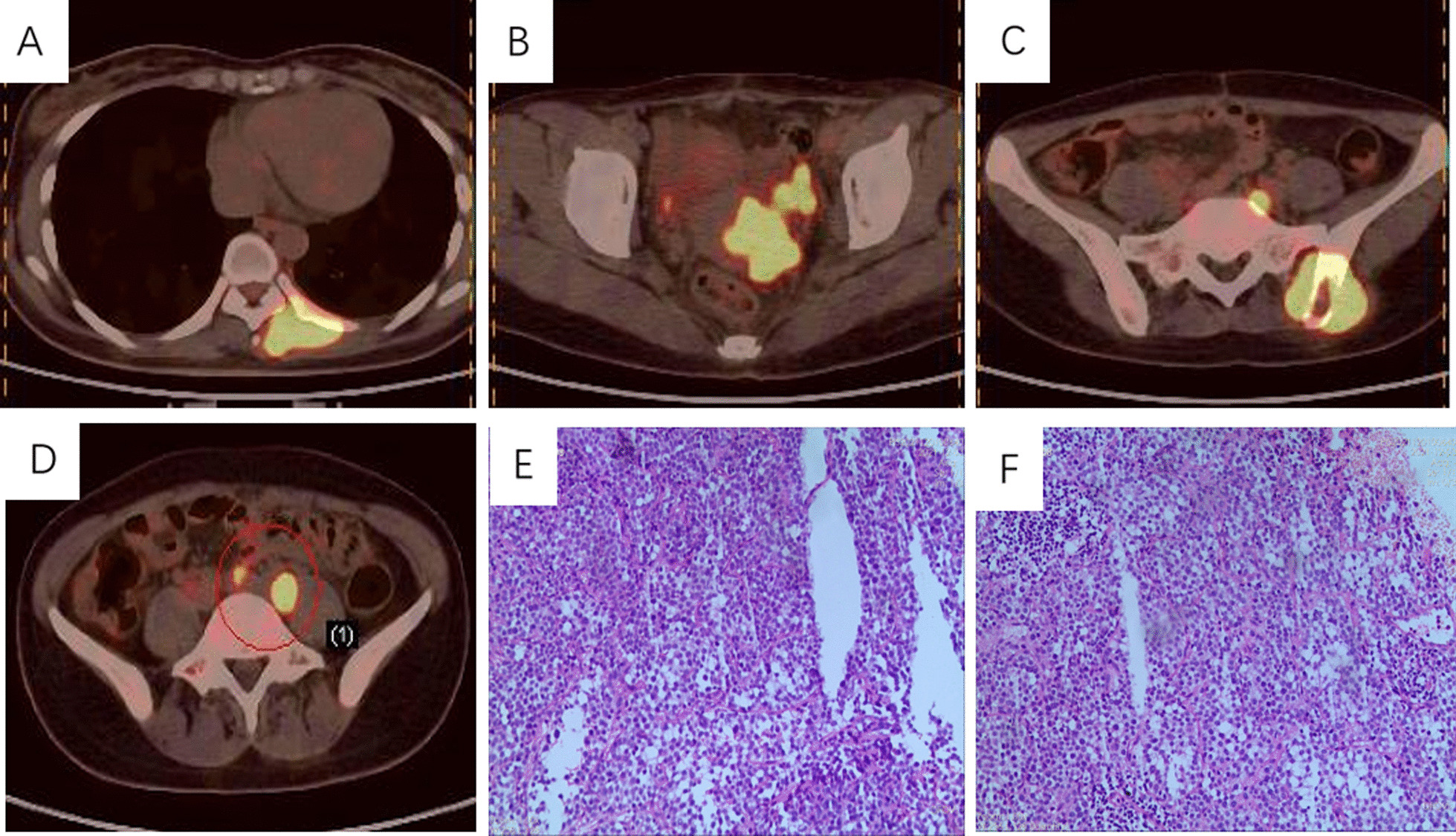
Multiple metastases were demonstrated by a positron emission computed tomography scan at the erector spinae area adjoining T7–T8 vertebral column (**A**), cervical site (**B**), the left iliac bone (**C**), and the retroperitoneal and pelvic lymph nodes (**D**). Hematoxylin and eosin stain for histopathology of the cervical biopsy indicated poorly differentiated squamous cell carcinoma (**E**, **F**). The area circled in **D** represents “Anterior sacral and left paravascular iliac metastatic lymph nodes”

The patient is a 33-year-old Chinese woman who was diagnosed with International Federation of Gynecology and Obstetrics (FIGO) stage IVB cervical cancer in November 2021. Instead of vaginal bleeding or abnormal discharge, her clinical symptoms were unusual and included a bloating sensation in the anal area and pain in the left buttock. Both the ThinPrep cytologic test (TCT) and the human papilloma virus (HPV) test were declared negative by the local clinic and a colonoscopy was carried out, ruling out colonic or rectal abnormalities. Gynecological examination showed enlargement of the cervix uteri, with infiltration to both parametria, but not reaching up to the pelvic wall or involving the vagina. Histopathology of the cervical biopsy indicated poorly differentiated squamous cell carcinoma (SCC) (Fig. 1). Multiple metastases were demonstrated by pelvic magnetic resonance imaging (MRI) and positron emission computed tomography (PET–CT) (Fig. 1) at the erector spinae area adjoining T7–T8 vertebral column, the left iliac bone, and the retroperitoneal and pelvic lymph nodes. From December 2021 to April 2022, the patient was enrolled in the clinical trial of sintilimab combined with chemotherapy and radiotherapy for treatment of stage IV cervical cancer and underwent six cycles of immunotherapy and chemotherapy (sintilimab plus paclitaxel liposome and cisplatin, this clinical trial was registered at the Chinese Clinical Trial Registry (www.chictr.org.cn) with registration number ChiCTR2100045676). Treatment was well tolerated and led to a partial response (PR). The masses adjacent to the spine and iliac bone was largely reduced. Thus, radiotherapy of the metastatic residues was carried out and followed by radiotherapy to the primary tumor at the cervix uteri.

**Fig. 2:**
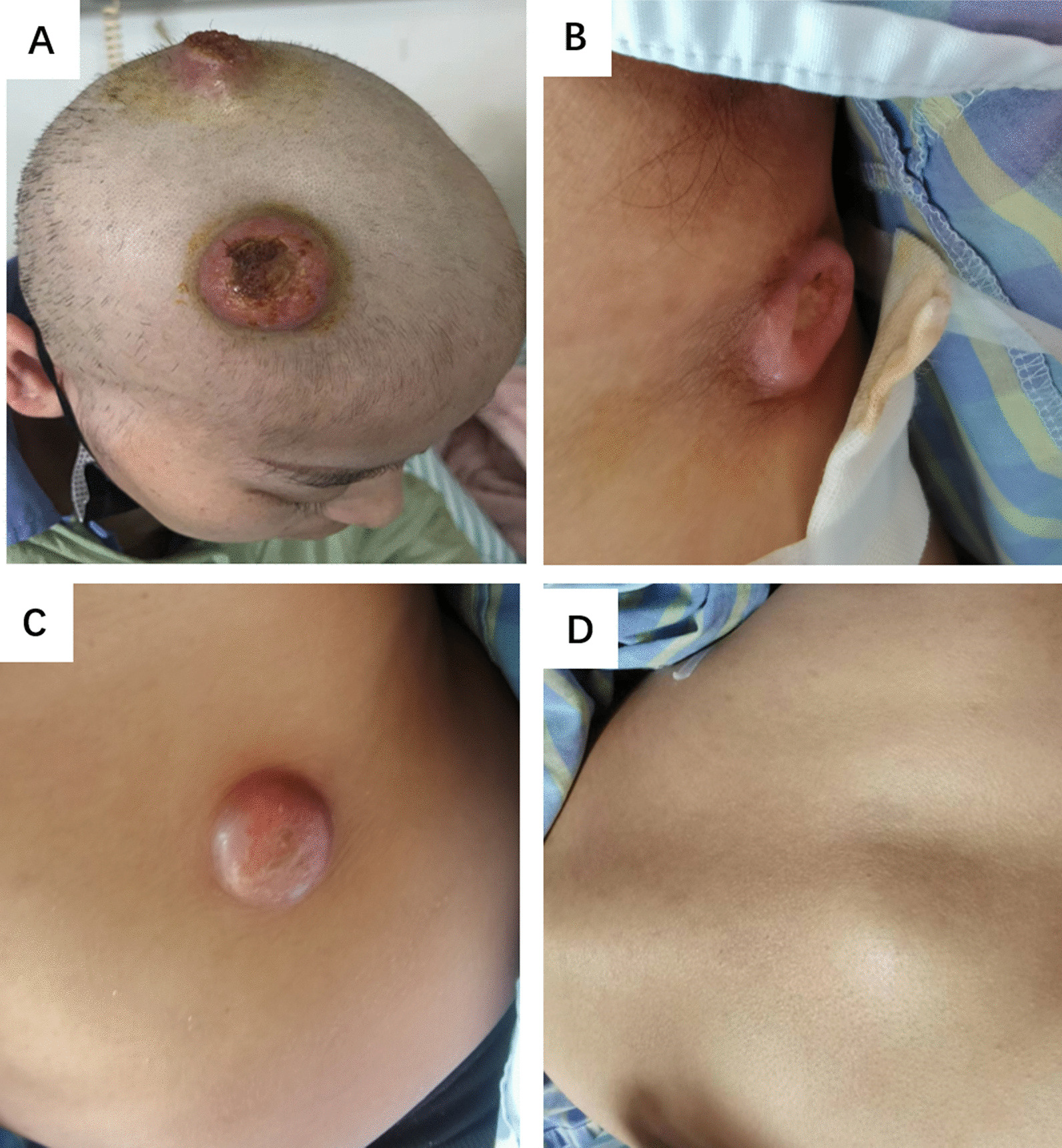
Subcutaneous metastasis on right frontal area, the top of the head (**A**), left axilla (**B**), right anterior abdomen (**C**), and left back (**D**)

**Fig. 3:**
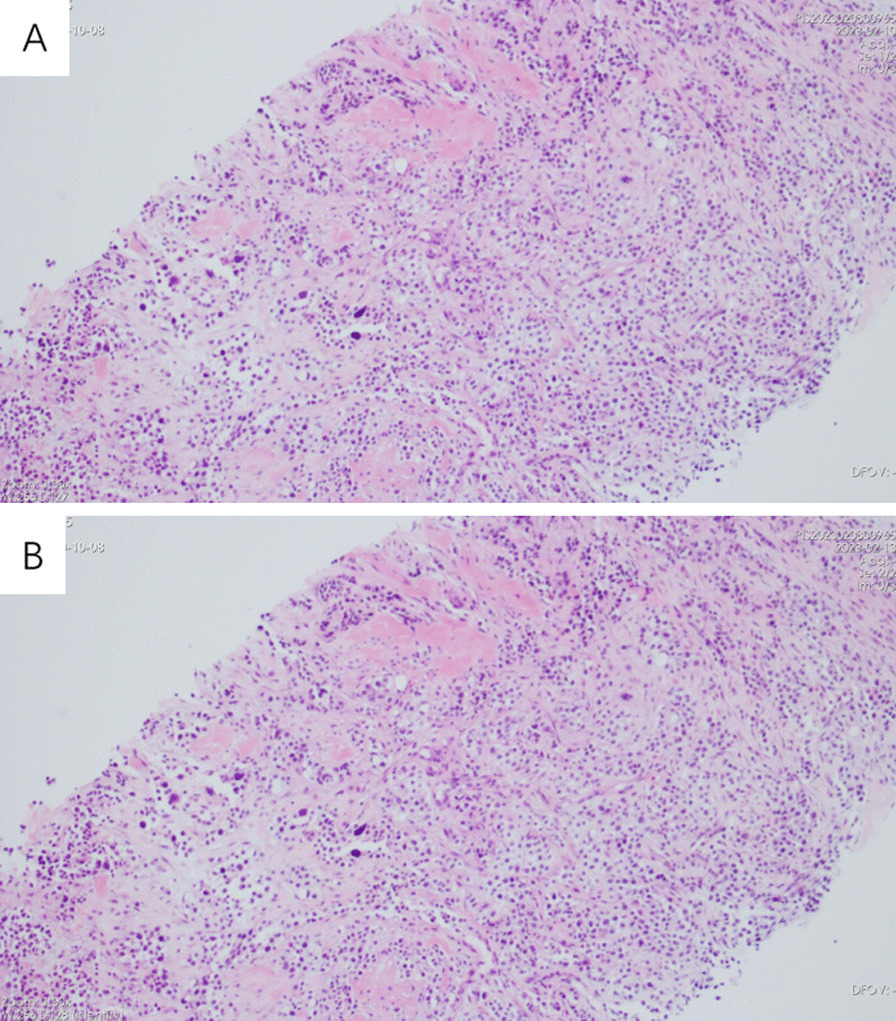
Hematoxylin and eosin stain for fine‑needle aspiration cytology of the left back swellings (**A**, **B**)

However, by the time of the radiotherapy completion in October 2022, the patient noticed painless nodules at the left scapular region and the right hypochondrium. The following month, more nodules occurred on the scalp and trunk including the left axilla, anterior abdomen, and left back, along with a lesion invading the sternum which caused acute bone pain (Fig. 2). The cutaneous masses were white, discrete with a rubbery consistency, and fixed to the skin. Several nodules increased in size and eventually ulcerated. Fine‑needle aspiration cytology (FNAC) of the left back swellings revealed metastatic squamous cell carcinoma, P16 stained positive (Fig. 3). No visceral or brain metastasis was observed through thoracic and abdominal CTs or head and pelvic MRIs at this point. The patient was given palliative chemotherapy due to the wide range of metastasis. The masses were temporarily reduced but then increased in scale and number again. The treatment was subsequently switched to cadonilimab, a programmed death 1/cytotoxic T-lymphocyte antigen 4 (PD-1/CTLA-4) bi-specific antibody recently approved for metastatic or recurrent cervical carcinoma.

## Discussion

Cutaneous metastasis is rare in cervical carcinoma and portends a fatal outcome. The average time from metastasis to death is 8.5 months [19]. It typically occurs in cases of tumor recurrence, but it has also been reported as part of the primary disease [20]. The average time between initial diagnosis and skin metastatic ranges from 16.9 to 20.7 months [19]. A review of 1190 patients revealed that the incidence of skin metastasis in stage 1 is 0.8%, with 1.2% in stages 2 and 3 both, and 4.8% in stage 4 [21]. Our patient first presented with cutaneous nodules 1 year after her first diagnosis.

Cutaneous metastases can present as nodules, plaques, or inflammatory telangiectatic lesions [22]. Nodules were the main form [21]. Other clinical manifestations includes maculopapular lesions, scar infiltration, and neoplastic alopecia [23].

While many exceptions do exist, it is true that metastases tend to occur on cutaneous surfaces in proximity of the primary tumor [6, 24]. Cervical cancer has a tendency to spread via lymphatics, and with evidence such as tumor cells plugging the lymphatic channels [25], it is assumed that the cutaneous metastases are a result of retrograde spread of the tumor secondary to lymphatic obstruction [22]. As for remote metastasis, hematogenous dissemination seems the reasonable explanation. Pertzborn *et al*., describing the metastases at the site where an intravenous catheter was placed, previously further confirmed this hypothesis [20, 26].

The scalp metastasis may be due to hematogenous spread via the external carotid artery branches and Batson’s plexus, a route that bypasses the pulmonary circulation [8, 13, 18]. Fay *et al*. suggested that the scalp afforded a high degree of vascularity, immobility, and warmth, which might enhance the proclivity for metastasis to this region [27].

In men, the primary tumor with scalp metastasis was often in the lung or kidney, while in women, the breast was the most frequent primary site [6]. However, scalp metastasis from cervical cancer is exceptionally rare, and the well-documented cases are summarized in Table 1 [7–18].

**Table1:** Summary of clinical and pathological features of patients with scalp metastasis from uterine cervical cancer

No.	First author	Year	Age	Histopathology	Primary staging	Skin metastatic site	Other metastasis	Time to metastasis	Morphology	Size (cm)	No. of lesions	Treatment for M	Survival
1	Shimizu, I. [16]	1983	59	SCC	IIIB	Scalp	Bones, para-aortic LN	6 months	Nodules	1	2	Chemo	3 months
2	Fogaca, M.F. [11]	1993	33	NEC	NS	Scalp, chest, back, abdomen, axilla, neck	NS	2 weeks	Nodules	0.2–1.2	Multiple	NS	NS
3	Maheshwari, G.K. [13]	2001	45	SCC	IIB	Scalp	None	10 months	Nodules	1–4	4	p R	Lost
4	Agarwal, U. [8]	2002	60	SCC	IIIB	Scalp	None	3 months	Lump	5 × 5	1	p R	NS
5	Park, J.Y. [15]	2002	47	SCC	IB	Scalp	Supraclavicular LN, pelvic LN, thoracolumbar, vertebral bodies, lung, liver	5 years	Nodules	2 × 2	1	p R + chemo	NS
6	Chung, J.J. [10]	2007	45	SCC	IB1	Scalp	Para-aortic LN, liver, pancreatic head, lumbar spine	8 years	Plaque, alopecia	5 × 2.5; 2 × 1.5	2	None	3 months
7	Chen, C.H. [9]	2007	72	SCC	IIB	Scalp, extremities, trunk	Lung, bone, brain	1 year	Papules and nodules	NS	Multiple	p chemo	6 months
8	Abhishek, A. [7]	2008	53	ADENO	IIA	Scalp	None	9 months	Lump	8 × 8	1	Chemo + p radio	Lost
9	Takagi, H. [17]	2010	48	SCC	IIB	Scalp	None	1 year	Swelling	2 × 2	1	p R	NS
10	Vitorino-Araujo, J.L. [18]	2013	55	SCC	IIIB	Scalp	NS	NS	Lump	10.5 × 7.0	1	Surgery	NS
11	Kuhn, T. [12]	2016	50	SCC	IVB	Scalp	Lung, LN, bone, brain	Simultaneously	Nodules	1–2	4	p chemo + p R	NS
12	Devnani, B. [31]	2018	45	NEC	IIB	Scalp, anterior abdomen, upper back	Liver	10 months	Nodules	1–2	Multiple	NEC	Chemo

*SCC* squamous cell carcinoma, *ADENO* adenocarcinoma, *NEC* neuroendocrine carcinoma, *chemo* chemotherapy, *p R* palliative radiotherapy

The average age of patients was 51 years. In only one case, scalp metastasis was presented as the initial manifestation of cervical cancer [12]. Occurrence of scalp metastases does not appear to be related to the stage of disease. Interval from initial diagnosis to development of scalp metastases varied from 2 weeks to 8 years. The main morphology were nodules and plaques with or without alopecia. The location and number of lesions were varied and nonuniform. Simultaneous metastatic involvement of other sites can be seen in most of the cases. Squamous cell carcinoma was the most prevalent histopathology type, partially because its dominating proportion in cervical cancer, and that in general, the metastatic lesion’s histology showed strong association to the primary ones [28].

Cutaneous metastatic nodules were often misdiagnosed as epidermoid cysts and less frequently as fibromas, papillomas, lipomas, or neurofibromas [28]. With the increasing utilization of immunotherapy in recurrent and advanced cervical cancer, more attention should be drawn to ruling out cutaneous metastases for patients who developed dermatologic toxicity from immune checkpoint inhibitors [29].

There is no clear guideline regarding the skin metastases treatment, and the main strategy usually remains palliative rather than curative [19]. Some cases suggest that with oligometastases, surgical resection combined with chemoradiation may prolong disease-free survival [29].

Skin metastasis from an underlying cancer is typically a preterminal occurrence. Although survival of up to 3 years has been documented, in most instances it is measured in weeks or months. Longer survival times and better cancer control may be achieved from early detection. Despite the rarity of cutaneous metastasis, all patients should have a comprehensive skin examination during routine appointments, with a low threshold for skin biopsy [30].

## Conclusions

Here, we report a case of cervical carcinoma with multiple metastases to the scalp and the trunk for the first time in mainland China in the literature. Cervical cancer metastases to the scalp are extremely uncommon. When a scalp metastasis is present, it might be the only symptomatic sign of disease progression or widespread metastatic lesions. So far, there is no clear guideline regarding skin metastases treatment. Such skin lesions warrant a thorough radiologic and pathologic workup to form a comprehensive management plan.

Time to metastasis refers to time from initial diagnosis to skin metastasis; Survival refers to the time from the occurrence of skin metastasis to death.

## Data Availability

The datasets used and/or analysed during the current study available from the corresponding author on reasonable request.

## Abbreviations

CC: Cervical cancer
TCT: ThinPrep cytologic test
HPV: Human papilloma virus
MRI: Magnetic resonance imaging
PET–CT: Positron emission computed tomography
FNAC: Fine‑needle aspiration cytology
HE: Hematoxylin and eosin
PD-1: Programmed death 1
CTLA-4: Cytotoxic T-lymphocyte antigen 4
